# A Case of *Magnusiomyces capitatus* Peritonitis Without Underlying Malignancies

**DOI:** 10.1177/2324709618795268

**Published:** 2018-08-21

**Authors:** Carlos D’Assumpcao, Benson Lee, Arash Heidari

**Affiliations:** 1 Ross University, Miramar, FL, USA; 2 Kern Medical—University of California Los Angeles, Bakersfield, CA, USA

**Keywords:** *Magnusiomyces capitatus*, *Geotrichum capitatum*, *Dipodascus capitatus*, *Trichosporon captiatum*, *Saprochaete capitata*, *Blastoschizomyces capitatus*, peritonitis

## Abstract

*Magnusiomyces capitatus* is a rare cause of fungal infection in immunocompromised patients, mainly seen in hematological malignancies. *M capitatus* infections are extremely rare in immunocompetent patients, as it is part of normal human microbial flora. We are presenting an extremely rare case of *M capitatus* peritonitis in an otherwise immunocompetent patient who suffered from gastrointestinal leakage due to pancreatitis. Fungal identification was performed at reference laboratory by phenotypic characteristics and DNA sequencing of target internal transcribed spacer region of the rRNA gene and the D1-D2 domain of the large-subunit rRNA gene and susceptibility testing by Clinical and Laboratory Standards Institute guidelines (document M27-S4) broth dilution method. He was successfully treated with a combination of surgical repair and voriconazole single therapy.

## Introduction

*Magnusiomyces capitatus*, previously known as *Geotrichum capitatum, Dipodascus capitatus, Trichosporon captiatum, Saprochaete capitata*, or *Blastoschizomyces capitatus*,^1^ is a rare cause of fungal infection in immunocompromised patients, mainly seen in hematological malignancies.^2-17^ *M capitatus* is extremely rare in immunocompetent patients, as it is part of normal human microbial flora.^18^ Presented here is a case of peritonitis infection with *M capitatus* without underlying malignancies.

## Case Report

A 32-year-old alcoholic male with liver steatosis presented with hemorrhagic necrotizing pancreatitis with peritonitis and retroperitoneum involvement. He was started on conservative therapy and percutaneous irrigation and drainage. Unfortunately, he rapidly deteriorated on hospital day 4 into acute abdominal compartment syndrome with acute respiratory distress. He was intubated and underwent damage control laparotomy resulting in pancreatic necrosectomy with subtotal pancreatectomy, splenectomy, repair of superior mesenteric vein, and wedge liver biopsy. Intraoperatively, peripancreatic necrosis was noted to extend proximally to diaphragm with extensive dissection throughout the retroperitoneum and at the root of the small bowel retroperitoneal area. During his second relaparotomy on hospital day 5 for removal of abdominal packing, incidental duodenal and gastric enterotomies were noted and repaired. Retroperitoneal edema was much improved. Cholecystectomy was performed for eosinophilic cholecystitis. Large Davol sump drains were placed for postoperative irrigation. Whittman patch and wound vacuum-assisted closure were placed. He required prolonged intensive care unit (ICU) admission with mechanical ventilation. Four additional operations were required to reapproximate his abdominal fascia. Skin was eventually closed on hospital day 17.

His course was also complicated by pleural effusions, pulmonary embolism, and persistent fevers and leukocytosis. Pleural effusions were therapeutically drained and were culture negative. Heparin was bridged to warfarin for his pulmonary embolism. Meropenem, linezolid, and micafungin were started empirically on hospital day 19.

**Figure 1.**
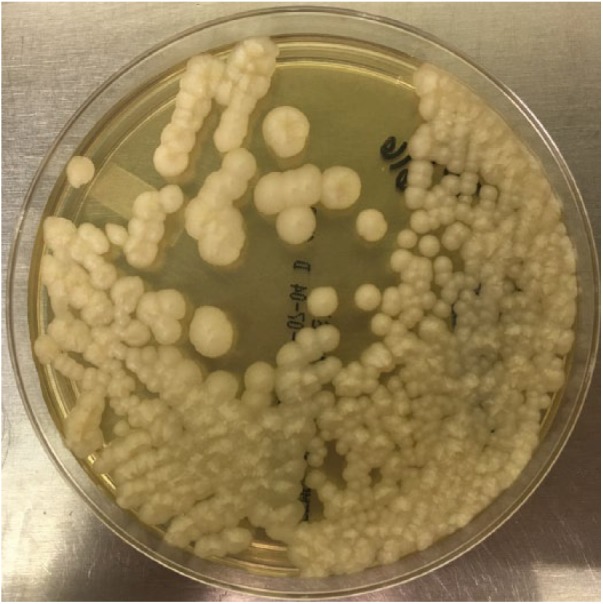
*Magnusiomyces capitatus*, peritoneal fluid, 5-day culture on Sabouraud Dextrose Agar, Emmons media (Thermo Scientific, Remel, Lenexa, KS).

**Figure 2.**
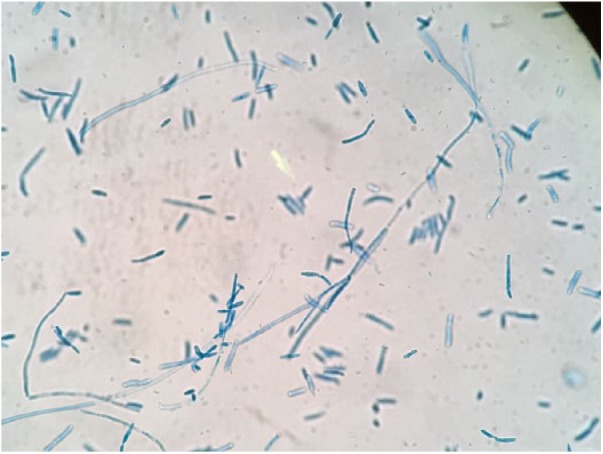
*Magnusiomyces capitatus*, peritoneal fluid, 2-day culture, lactophenol cotton blue stain, 40× magnification.

**Figure 3.**
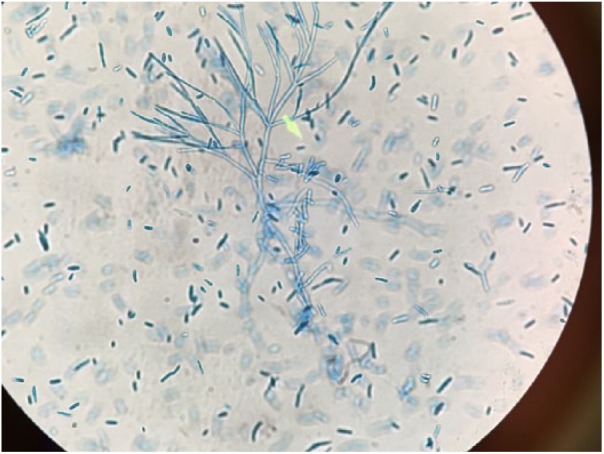
*Magnusiomyces capitatus*, retroperitoneal fluid, 7-day culture, lactophenol cotton blue stain, 40× magnification.

Peritoneal fluid was collected on hospital day 19 and sent for culture, which grew *Klebsiella oxytoca* and vancomycin-resistant *Enterococcus faecium* (VITEK2, bioMérieux, Durham, NC). There was suspicion of incomplete drainage of intraabdominal fluid, and so a retroperitoneal drain was placed by interventional radiology on hospital day 31. Culture of this retroperitoneal fluid grew vancomycin-resistant enterococci *E faecium* (VITEK2, bioMérieux) and *M capitatus* (identification by phenotypic characterization and DNA sequencing of targets internal transcribed spacer region of the rRNA gene and the D1-D2 domain of the large-subunit rRNA gene and the D1-D2 domain of the large-subunit rRNA gene by University of Texas Health Science, San Antonio, TX; see Figures 1-3). Peritoneal fluid was collected again from hospital day 40, and it grew *M capitatus, K oxytoca*, and *Streptococcus sanguinis* (VITEK2, bioMérieux). He also developed eosinophilia (absolute eosinophil count of 800) on hospital days 42 to 46.

Meropenem was de-escalated to a 2-week course of ceftriaxone on hospital day 45 (changed to ciprofloxacin at discharge). Linezolid was discontinued after a 2-week course was completed. A 12-week course of voriconazole (minimum inhibitory concentration = 0.25 µg/mL by Clinical and Laboratory Standards Institute broth dilution M27-S4 method by the University of Texas Health Science, San Antonio, TX; see Table 1) was started on hospital day 45. Warfarin for his pulmonary embolism was switched to enoxaparin due to drug-drug interaction of warfarin with voriconazole. He started to improve and was eventually discharged home on hospital day 50 with follow-up in outpatient clinic, ambulating and tolerating food.

**Table 1.** *Magnusiomyces capitatus*, Retroperitoneal Fluid, Antifungal Sensitivities by the University of Texas Health Science, San Antonio, TX, by the Clinical and Laboratory Standards Institute Broth Dilution (M27-S4) Method. No Established Break Points.

Antifungal Agent	Minimum Inhibitory Concentration
Amphotericin B	1 µg/mL
Natamycin	8 µg/mL
Fluconazole	8 µg/mL
Itraconazole	1 µg/mL
Posaconazole	0.5 µg/mL
Voriconazole	0.25 µg/mL
Isavuconazole	0.25 µg/mL

At 12-week follow-up, the patient reported abstinence from alcohol since initial hospital admission. The patient’s wife was supportive during the entire hospital stay as well as the post hospital recovery, ensuring wound dressing changes and medication compliance. Liver function was monitored every 3 to 4 weeks as an outpatient throughout the 12-week course of voriconazole. Liver function was within normal limits. He completed a 90-day course of anticoagulation.

## Discussion

To our knowledge, this is an extremely rare case of *M capitatus* peritonitis in an otherwise immunocompetent patient who suffered from gastrointestinal leakage due to pancreatitis, likely from the gastric and duodenal enterotomies found and repaired on hospital day 5. He was successfully treated with a combination of surgical repair and voriconazole.

Literature review suggests an intrinsic resistance to echinocandins^19^; however, in vitro and in vivo activity of antifungals may differ. Liposomal amphotericin B and azoles, specifically voriconazole and posaconazole, have had reported clinical success.^7,16,20^ In vitro studies with flucytosine, fluconazole, and itraconazole showed poor susceptibilities.^21^ No susceptibility break points have been determined yet.

The newest triazole, isavuconazole, demonstrated excellent in vitro activity against *M capitatus*.^22^ In the SECURE trial, a phase 3, randomized, controlled, noninferiority clinical trial against aspergillus and other filamentous fungi, isavuconazole was equally tolerable but had better pharmacokinetics and fewer drug-related adverse events compared with voriconazole.^23^ Due to identical minimum inhibitory concentration of our patient’s isolate to voriconazole and isavuconazole (see Table 1), voriconazole was selected as the initial triazole antifungal therapy so that isavuconazole could be reserved for rescue therapy in the event that voriconazole did not improve clinical status. Recently, ICU admissions have been linked to the development of *M capitatus* infection. In Italy, a non-neutropenic patient in the ICU after cardiac surgery developed *M capitatus* fungemia.^24^ In Croatia, a fatal *M capitatus* respiratory tract infection was diagnosed posthumously in a patient who became febrile 7 days into his ICU admission for polytrauma.^25^ Moreover, a recent survey of *M capitatus* infections in the ICU and hematology-oncology unit within a single hospital in Turkey found the strains to be genetic clones. However, microbiological investigations of the hospital environment failed to find the isolate.^26^ While *M capitatus* is considered a ubiquitous environmental organism and part of the normal human gastrointestinal flora,^18^ to the authors’ knowledge, there has not been any case reports or studies tracing *M capitatus* to a hospital fomite. More studies are needed to determine a true correlation between ICU admissions and *M capitatus* infections.

Vancomycin-resistant *E faecium, S sanguinis*, and *K oxytoca* likely had a gastrointestinal instead of a cutaneous source. While coinfection may have caused peritonitis in this patient, his clinical status did not improve until the addition of the appropriate antifungal covering this particular strain of *M capitatus*.
